# Gastrointestinal Distension by Pectin-Containing Carbonated Solution Suppresses Food Intake and Enhances Glucose Tolerance *via* GLP-1 Secretion and Vagal Afferent Activation

**DOI:** 10.3389/fendo.2021.676869

**Published:** 2021-06-08

**Authors:** Kento Ohbayashi, Yukiko Oyama, Chiharu Yamaguchi, Toshiki Asano, Toshihiko Yada, Yusaku Iwasaki

**Affiliations:** ^1^ Laboratory of Animal Science, Graduate School of Life and Environmental Sciences, Kyoto Prefectural University, Kyoto, Japan; ^2^ Self-Medication R&D Laboratories, Taisho Pharmaceutical Co., Ltd., Saitama, Japan; ^3^ Division of Integrative Physiology, Kansai Electric Power Medical Research Institute, Kobe, Japan; ^4^ Division of Diabetes and Endocrinology, Kobe University Graduate School of Medicine, Kobe, Japan

**Keywords:** gastric distension, intestinal distension, glucagon-like peptide-1, vagal afferents, food intake, insulin sensitivity, obesity, feeding rhythm

## Abstract

Diet-induced gastrointestinal distension is known to evoke satiation and suppress postprandial hyperglycemia; however, the underlying mechanisms remain poorly understood. This study explored how gastrointestinal distension regulates energy homeostasis by using inflating stomach formulation (ISF), the carbonated solution containing pectin that forms stable gel bubbles under acidic condition in the stomach. Here we show that, in mice, oral administration of ISF induced distension of stomach and proximal intestine temporarily, stimulated intestinal glucagon-like peptide-1 (GLP-1) secretion, and activated vagal afferents and brainstem. ISF suppressed food intake and improved glucose tolerance *via* enhancing insulin sensitivity. The anorexigenic effect was partially inhibited, and the beneficial glycemic effect was blunted by pharmacological GLP-1 receptor blockade and chemical denervation of capsaicin-sensitive sensory nerves. In HFD-fed obese mice showing arrhythmic feeding and obesity, subchronic ISF treatment at the light period (LP) onset for 10 days attenuated LP hyperphagia and visceral fat accumulation. These results demonstrate that gastrointestinal distension by ISF stimulates GLP-1 secretion and the vagal afferent signaling to the brain, thereby regulating feeding behavior and glucose tolerance. Furthermore, subchronic ISF treatment ameliorates HFD-induced visceral obesity. We propose the diet that induces gastrointestinal distension as a novel treatment of hyperphagic obesity and diabetes.

## Introduction

Glucagon-like peptide-1 (GLP-1) is a peptide hormone produced primarily in the intestinal endocrine L cells and additionally in the brainstem. Secretion of intestinal GLP-1 is triggered by luminal macronutrient and plant polyphenols within 15–30 min after meal (1). The meal-evoked GLP-1 secretion regulates postprandial functions such as satiation and blood glucose disposal (2). In the current situation with increasing prevalence of obesity and type 2 diabetes globally, GLP-1 receptor (GLP-1R) agonists show remarkable therapeutic effects (2, 3) and attract attention. GLP-1R agonists, unlike endogenous intestinal GLP-1, are stable and have a longer half-life in the circulation and hence, act directly on pancreatic *β* cells (4) and hypothalamic neurons regulating glycemia and/or feeding (5, 6), thereby ameliorating hyperglycemia and overeating through direct action of GLP-1R agonists on target organs such as pancreatic *β* cells and central nerves (7, 8). However, GLP-1R agonists also elicit adverse effects including nausea and vomiting (9, 10), the effects possibly exerted by their passage through the blood–brain barrier to directly act on neurons in the brain (5, 6).

Vagal afferent neurons have cell bodies in nodose ganglion (NG) and bipolarly project to the peripheral organs and the nucleus tractus solitarius (NTS) in the brainstem. Thus, vagal afferents play a role in sensing several mechanical and chemical signals in the periphery, converting them to the neuronal information and conveying it to the NTS (11, 12). Vagal afferents directly sense postprandial gut/pancreatic hormones, such as cholecystokinin (CCK), peptide YY (PYY), nesfatin-1, oxytocin, and insulin, and thereby regulate food intake (13–18). Recent studies have indicated that intestinal GLP-1 increases insulin secretion and suppresses food intake through a vagal afferent neural pathway (19, 20). Furthermore, we have recently shown that a rare sugar D-allulose induces intestinal GLP-1 secretion and consequent activation of vagal afferents, thereby inhibiting food intake and promoting glucose tolerance by enhancing insulin release and action (21). Therefore, vagal afferents mediate the beneficial feeding and metabolic effects of intestinal GLP-1.

Gastric distension is reportedly one of factors involved in the termination of feeding (22). Consistently, taking bulky foods with low energy-density and high-volume, such as salad, at the beginning of a meal induces satiation and suppresses postprandial rises in blood glucose (23–25). Thus, similar effects are induced by gastric distension and GLP-1. It has recently been shown that an increase of gastric capacity using an inflating balloon promotes GLP-1 secretion in anesthetized rats (26). Besides, it is shown by *in vivo* calcium imaging that gastric distension activates the subclass of vagal afferent nerves expressing GLP-1R (27). However, it is unknown whether gastric distension regulates feeding and glucose metabolism *via* GLP-1 secretion and its interaction with vagal afferents.

The present study explored whether gastrointestinal distension suppresses food intake and promotes glucose tolerance *via* stimulating GLP-1 secretion and sensory nerves including vagal afferents. The method of gastric distension using intragastric balloon inflation has been used in numerous previous experiments. However, this method restricts the experimental protocol to a larger size of experimental animals and use of anesthesia (26–30). In the present study, to expand upper gastrointestinal tract we used the carbonated solution containing low methoxyl (LM) pectin, named as inflating stomach formulation (ISF), which forms stable gel bubbles under acidic condition in the stomach and inflates the stomach in humans (**Table 1**) (31). We firstly examined whether ISF induces gastrointestinal distension, GLP-1 secretion, and vagal afferent activation. Secondly, we explored whether these factors participated in the suppression of feeding and promotion of glucose tolerance using GLP-1R antagonists, chemical denervation of capsaicin-sensitive sensory nerves including vagal afferents. We found that oral administration of ISF expands the upper gastrointestinal tract, promotes GLP-1 secretion, and activates vagal afferents. Our data also support an essential role of the GLP-1-sensory nerve signaling pathway in the ISF action to reduce food intake and improve glucose tolerance *via* enhancing insulin sensitivity. In obese mice fed a high-fat diet exhibiting arrhythmic hyperphagia, subchronic administration of ISF corrects arrhythmic overeating and ameliorates visceral fat accumulation.

**Table 1:** Composition of ISF (inflating stomach formulation) and control solution (Control) and results of inflating rate of these solutions *in vitro*.

Component	unit	【ISF】	【Control】
1) Low methoxyl pectin	mg	1,850	0
2) Citric acid monohydrate	mg	200	200
3) Sodium benzoate	mg	92.5	92.5
4) Sodium hydroxide	mg	400	400
5) Non-carbonated water	ml	0	185
6) Carbonated water	ml	185	0
pH		3.8 ± 0.2	3.7 ± 0.02
Gas volume	GV	2.89	0
Inflating rate (after 10 min)	%	244.1 ± 3.2	100.0 ± 0.0

The preparation methods of ISF and control solution were described in Materials and Methods. Inflating rate was calculated from the results of in vitro inflating experiment (see Materials and Methods). In brief, ISF or control solution at 5°C was added to artificial gastric juice gently and was left to stand at 37°C for 10 min. The volume of solution and bubble-containing gel were measured using the scale of a quantitative measuring flask and then calculated as inflating rate.

## Materials and Methods

### ISF, Control Solution, and These Compositions

ISF and its control solution were prepared according to the methods reported by Domoto et al. with minor modifications (31). Citric acid monohydrate (200 mg), sodium hydroxide (400 mg), and LM pectin (1,850 mg) were mixed and dissolved in 43 ml of purified water. This solution was added with sodium benzoate as preservative and filled up to 185 ml with carbonated water (ISF, **Table 1**). The control solution for ISF did not contain LM pectin, and carbonated water was replaced with non-carbonated water (control solution, **Table 1**). These prepared solutions were filled in aluminum cans and stored at 5°C until the experiments.

### *In Vitro* Inflating Experiment of ISF and Control Solution

ISF, which is carbonated solution containing low methoxyl (LM) pectin, forms stable gel bubbles under acidic condition (31). The inflating rate of ISF and control solution *in vitro* was measured as described previously (31). ISF or control solution (185 ml, 5°C) was gently added to artificial gastric juice (Japanese Pharmacopoeia Disintegration Test Solution 1 (pH 1.2), 50 ml, 37°C) in a measuring flask. Then, this mixed solution was left to stand at 37°C. Ten minutes after addition, these total volumes including bubble-containing gal were recorded using the scale of the measuring flask. The inflating rate of the gel was calculated by the following formula; inflating rate (%) = {total volume after addition (X ml) – volume of artificial gastric juice (50 ml)}/sample volume (185 ml) * 100.

### Animals

Male C57BL/6J mice were purchased from Charles River Laboratory Japan (Yokohama, Japan) and housed for at least 1 week under conditions of controlled temperature (22.5 ± 2°C), humidity (55 ± 10%), and lighting (light period; LP 7:30–19:30). Mice were supplied standard chow (CE-2 with 3.4 kcal/g, 4.6% kcal from fat, 24.8% kcal from protein, and 49.9% kcal from carbohydrates, CLEA Japan, Tokyo, Japan) and water *ad libitum*. Diet-induced obese C57BL/6J mice (DIO mice, body weight; 35–45 g, age; 16–20 weeks-old) were produced by high-fat diet (HFD-60 with 4.9 kcal/g, 60.7% kcal from fat, 17.9% kcal from protein, and 21.4% kcal from carbohydrates, Oriental Yeast Co. Ltd., Tokyo, Japan) feeding for 50–100 days. Mice age 8–20 weeks were used and sufficiently habituated to handling before the experiments. Animal experiments were carried out after receiving approval from the Institutional Animal Experiment Committee of the Kyoto Prefectural University and in accordance with the Institutional Regulation for Animal Experiments.

### Measurement of GLP-1 in Portal Vein Plasma

Lean or DIO mice were fasted overnight (18:00 to next 10:00). ISF or control solution were po administered at 30 ml/kg (approximately 600 µl) at 10:00 in lean mice. Whereas, in the DIO mice (BW; approximately 40 g), control solution (15 ml/kg; 600 µl) or ISF (15 or 20 ml/kg: 600 or 800 µl) was administered. Then, the blood samples were collected from the portal vein under isoflurane anesthesia at 0, 1, 2, and 3 h after injection. The sampling syringe contained heparin (final concentration; 50 IU/ml), aprotinin (final concentration; 500 KIU/ml), and DPP-IV inhibitor vildagliptin (final concentration; 10 µM, for stable measurements). Plasma was collected after centrifugation (3,300 rpm, 10 min at 4°C) and stored at −80°C until assay. Total GLP-1 levels were measured using GLP-1 total ELISA kit (EZGLP1T-36K; Millipore, MA, USA).

### Systemic Capsaicin Treatment

To impair capsaicin-sensitive sensory nerves, systemic capsaicin treatment was performed as described (17). In brief, mice were anesthetized with tribromoethanol (200 mg/kg, ip), followed by subcutaneous (sc) administration of capsaicin at 50 mg/kg (5 ml/kg, solution composition: 10% ethanol, 10% Tween80, and 80% saline) (Day 1). A second capsaicin (50 mg/kg, sc) injection was performed at Day 3 with the same protocol. Finally, at Day 5, capsaicin (10 mg/kg, 10 ml/kg, ip) was injected into the conscious mice.

### Immunohistochemical Detection of pERK1/2 in Nodose Ganglion and Medial Nucleus Tractus Solitarius

ISF (30 ml/kg) or control solution (30 ml/kg) was po administered in C57BL/6J mice and those systemically treated with capsaicin, both fasted 16 h. At 30 min after injection, mice were transcardially perfused with Zamboni solution (4% paraformaldehyde and 0.2% picric acid in 0.1 M phosphate buffer at pH 7.4) under anesthesia. The nodose ganglia (NGs) and brain were collected, postfixed in the same fixative for 2 and 4 h at 4°C, respectively, and incubated in phosphate buffer containing 30% sucrose for 48 h at 4°C.

Longitudinal sections (10 µm) of NGs were cut with 60 µm intervals using a precision cryostat (Leica Microsystems, IL). Coronal sections (40 µm) of the hindbrain were cut with 120 µm intervals using a freezing microtome (Yamato Kohki industrial Co. Ltd., Saitama, Japan). Rabbit polyclonal antibody against phospho-p44/42 MAPK (Thr202/Tyr204, pERK1/2) (1/500; #9101; Cell Signaling Technology, MA, USA) and Alexa 488-conjugated goat anti-rabbit IgG (1/500; A11008; Invitrogen, CA, USA) were used for detection of pERK1/2 in NG and medial NTS. Fluorescence images were acquired with AxioObserver Z1 microscope and Axiocam 506 color camera (Zeiss, Oberkochen, Germany). Neurons immunoreactive to pERK1/2 in medial NTS (bregma −7.32 to −7.76 mm) were counted an averaged per mouse. In NGs, a number of pERK1/2-positive NG neurons in four sections per mouse were counted and averaged.

### Measurement of Food Intake

The mice were housed in individual cages and sufficiently habituated to standard powdered diet (CE-2, CLEA Japan) in a feeding box (Shinano Manufacturing Co., Ltd., Tokyo, Japan) and to handling at least 1 week before experiments. The mice were deprived of food (16 h fasting, 18:00 to 10:00 next day) with free access to water before the feeding experiment. On the next day at 9:30, ISF (20 or 30 ml/kg) or control solution (30 ml/kg) was po administrated, and at 10:00, CE-2 powdered diet was given. Then food intake for the following 1, 2, 3, 6, 24, and 48 h was measured by subtracting uneaten food from initially premeasured food and checking the food spillage. The cumulative energy intake including food eaten (CE-2; 3.4 kcal/g) and injected ISF (pectin; 2 kcal/g) in each time was expressed. To examine the involvement of GLP-1R signaling, exendin(9–39) amide [Ex(9–39), ab141101, Abcam, Cambridge, UK] at 600 nmol/kg or saline (5 ml/kg) was ip administered with a single shot at 15 min before ISF po administration.

### Conditioned Taste Aversion Test

Conditioned taste aversion test was performed as previously reported (17). To accustom mice to water deprivation schedule, mice were allowed access to two water bottles for 2 h (10:00−12:00) for 5 days. On the 6th day, mice were given 0.15% saccharine instead of water for 0.5 h, and then injected with control solution (30 ml/kg, po), ISF (30 ml/kg, po), or lithium chloride (LiCl, 3 mmol/kg, 20 ml/kg, ip), as a positive control to cause taste aversion (32). The 7th day was the rest day when mice had free access to normal water for 2 h. On the 8th day, two-bottle preference (0.15% saccharine *vs*. water) test was performed for 0.5 h. Conditioned taste aversion was determined as saccharine preference ratio [intake of saccharine solution (g)/intake of saccharine solution and water (g)].

### Glucose Tolerance Test and Insulin Tolerance Test

Mice were fasted overnight (18:00 to next 10:00 in lean mice and capsaicin-treated mice) or for 4 h (9:00 to 13:00 in DIO mice) in glucose tolerance test and for 4 h (9:00 to 13:00) in insulin tolerance test. Then 30 ml/kg ISF or control solution in lean and capsaicin-treated mice and 15 ml/kg ISF or control solution in DIO mice were po administered at 60 min prior to glucose and insulin tolerance test. Blood was collected from the tail vein at 0 min, followed by ip injection of D-glucose (1 or 2 g/kg) or insulin (1 IU/ml, porcine insulin from sigma I5523), and blood samples were collected at 15–240 min from the tail vein using heparinized capillary glass. Glucose levels in the blood samples were determined by GlucoCard Plus Care (Arkray, Kyoto, Japan). Plasma insulin was determined by insulin ELISA kit (Morinaga, Yokohama, Japan). Ex(9-39) (600 nmol/kg, 5 ml/kg) was ip administered with a single shot at 15 min before ISF injection.

### Daily Administration of ISF in HFD-Fed Obese Mice

DIO mice fed a HFD for 8 weeks (16 weeks-old) were po administered with ISF (20 ml/kg: 800 µl/mouse) or control solution (20 ml/kg: 800 µl/mouse) once daily at light period onset (7:30) for 10 days. Lean mice fed a standard chow (CE-7 with 3.4 kcal/g, 4.0% kcal from fat, 18.2% kcal from protein, and 57.1% kcal from carbohydrates, CLEA Japan) aged 16 weeks without po injection were used as the control. Body weight and food intake at light period (LP) onset and dark period (DP) onset were measured every day. On the 11th day, food intake was deprived at 7:30, and mice were anesthetized by isoflurane inhalation at 13:30. Blood and organ samples (liver, mesenteric, perirenal and epididymal white adipose tissue, intrascapular brown adipose tissue) were collected, and their wet organ weights measured, and then stored at −80°C until assayed. Insulin resistance was assessed using HOMA-IR as follows (33); [insulin (ng/ml) × 26 × blood glucose (mg/dl)/405] index. To measure triacylglycerol (TG), dissected liver cubes were extracted using chloroform/methanol (2:1) for 48 h at room temperature with shading, organic solvents were evaporated under N_2_ stream, and the crude lipids were re-suspended in isopropanol. TG concentrations in the solution were measured by TG specific enzymatic kit (Wako, Osaka, Japan). TG contents were normalized to the weight of liver.

### Statistical Analysis

All data were shown as means ± SEM. Statistical analysis was performed by two-tailed unpaired t-test or by one-way or two-way ANOVA. When ANOVA indicated a significant difference among groups, these groups were compared by Dunnett’s, Tukey’s or Bonferroni’s *post hoc* test. All statistical analyses were performed using Prism 7 (GraphPad Software, CA). p <0.05 was considered significant.

## Results

### Peroral ISF Expands Upper Gastrointestinal Tract and Promotes GLP-1 Secretion

**Figure 1:**
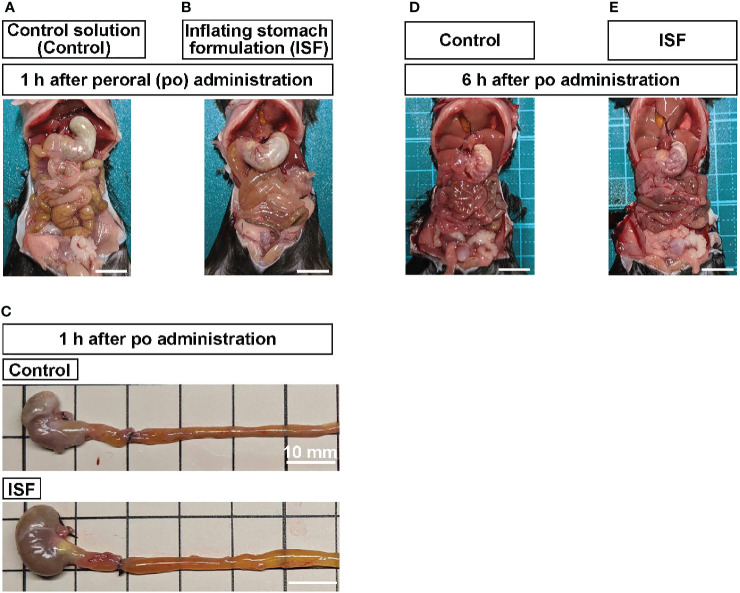
Peroral (po) administration of inflating stomach formulation (ISF) induces distension of gastrointestinal tract in mice. **(A, B)** Gastric and intestinal distension at 1 h after po injection of control solution (30 ml/kg, **A**) or ISF (30 ml/kg, **B**) in mice fasted overnight. **(C)** Stomach and intestine collected at 1 h after administration. To maintain the distension, the gastric cardia, the joining part of common bile duct on the duodenum, and middle part of ileum were tied with black strings. **(D, E)** At 6 h after injection, ISF-induced distension of stomach and intestine returned to the control level. Scale bar indicates 10 mm.

**Figure 2:**
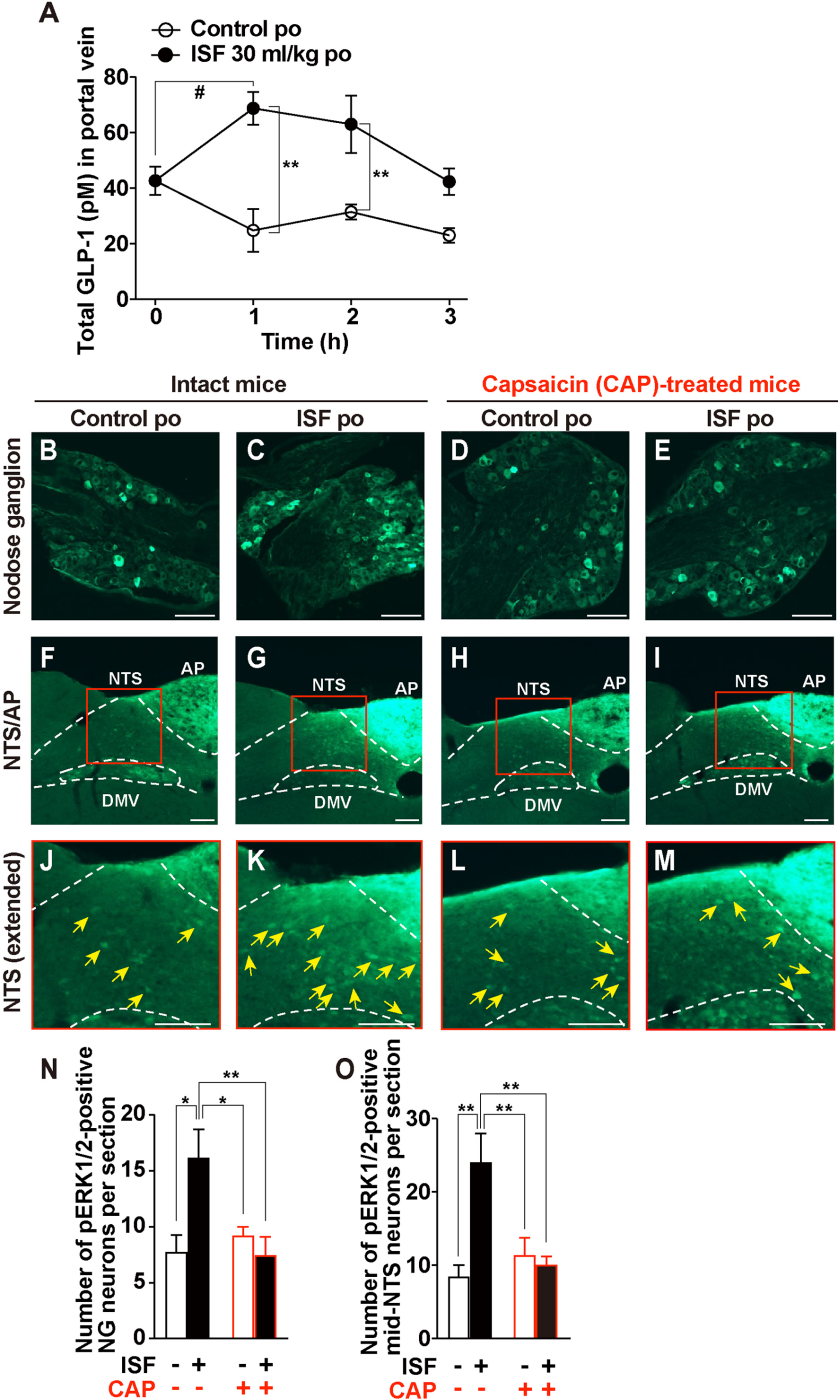
ISF promotes GLP-1 secretion and activates the nodose ganglion (NG) and the nucleus tractus solitarius (NTS) *via* capsaicin-sensitive sensory nerves. **(A)** Time course of total GLP-1 concentrations in portal vein plasma after po administration of 30 ml/kg ISF or control solution. *n* = 7. ^**^ *p* < 0.01 by two-way ANOVA followed by Bonferroni’s test *vs*. control. ^#^ *p* < 0.05 by two-way ANOVA followed by Dunnett’s test *vs.* 0 min. **(B–I)** NG **(B–E)** and NTS **(F–M)** sections immunostained for pERK1/2 at 30 min after po administration of 30 ml/kg ISF or control solution in control and systemic capsaicin (CAP)-treated mice. **(J–M)** Extended pictures of red squares in **(F–I)**, respectively. Arrowhead indicates pERK1/2-positive neurons. Scale bar, 100 µm. **(N, O)** Number of pERK1/2-positive neurons in the NG **(N)** and in the medial NTS **(O)**. CAP(−); intact mice. CAP(+); CAP-treated mice. *n* = 5–6. ^*^ *p* < 0.05 and ^**^ *p* < 0.01 by one-way ANOVA followed by Tukey’s test. Data show means ± SEM.

Previous study has shown that carbonated beverage containing LM pectin expands stomach in humans (31). In this study, the composition of ISF was modified to increase the inflating potency, so that the inflating ratio *in vitro* was approximately 240% compared with control solution (**Table 1**). We firstly examined whether ISF induces gastric distension in mice. Peroral (po) administration of ISF (30 ml/kg) into the stomach, using stainless steel feeding needle, expanded the upper gastrointestinal tract including the stomach, duodenum, and ileum at 1 h after injection (**Figures 1A–C**). Then, we examined whether gastrointestinal distension increases GLP-1 secretion. Total GLP-1 concentrations in the portal vein significantly increased at 1 and 2 h and returned to the basal level at 3 h after po ISF administration (**Figure 2A**).

### ISF Induces ERK1/2 Phosphorylation in Nodose Ganglion and Medial NTS

GLP-1 directly excites vagal afferent nodose ganglion (NG) neurons expressing GLP-1R (34, 35), and intestinal GLP-1 release activates vagal afferent nerves innervating intestine and portal/liver areas and projecting to medial NTS (21). We investigated whether po ISF, which induces GLP-1 secretion, increases expression of phosphorylated ERK1/2 as cellular/neuronal activation markers (36) in NG and medial NTS. ISF (30 ml/kg, po), compared to control solution, significantly increased pERK1/2 expression in both NG neurons and medial NTS at 30 min after injection (**Figures 2B, C, F, G, J, K, N, O**). Systemic capsaicin (CAP) treatment desensitizes capsaicin-sensitive sensory nerves including vagal afferents (37). The ISF-induced pERK1/2 expression in both NG and NTS was blunted in CAP-treated mice (**Figures 2D, E, H, I, L, M, N, O**). These data indicated that oral administration of ISF activates medial NTS neurons *via* activating vagal afferent neurons. In addition, po ISF increased pERK1/2-immunoreative fluorescence in the area postrema (AP) in both intact mice and CAP-treated mice (**Supplementary Figure 1**).

### ISF Dose-Dependently Suppresses Food Intake Without Aversive Behavior

**Figure 3:**
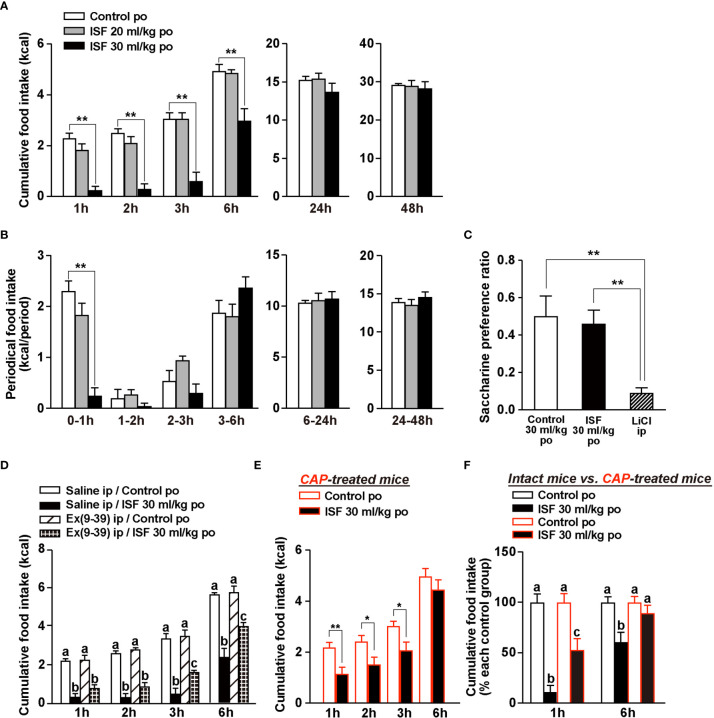
ISF suppresses food intake without aversive behavior in a manner partially dependent on GLP-1R signaling and capsaicin-sensitive sensory nerves. **(A, B)** Cumulative **(A)** and periodical **(B)** food intake at 1–48 h after po administration of ISF (20 or 30 ml/kg) or control solution in mice fasted overnight (16 h). *n* = 6. **(C)** In conditioned taste aversion test, saccharin preference was measured at 2 days after injection of control solution (30 ml/kg, po), ISF (30 ml/kg, po) or lithium chloride (LiCl; 3 mmol/kg, ip). *n* = 6–8. **(D)** Ip injection of 600 nmol/kg exendin(9–39) amide [Ex(9-39)], which is GLP-1R antagonist, attenuated the ISF-induced anorexigenic effects at 3 to 6 h but not at 2 h and earlier of injection. *n* = 4–6. **(E)** The action of po ISF (30 ml/kg) injection to inhibit food intake was partially attenuated at 1 to 3 h and blunted at 6 h of injection in systemic CAP-treated mice. *n* = 8. **(F)** Cumulative food intake [taken from panels **(A, E)**] with and without ISF was normalized to that without ISF (control solution) and expressed by percentage. ^**^ *p* < 0.01 by one-way ANOVA followed by Dunnett’s test *vs.* control solution **(A, B)**, ^**^ *p* < 0.01 by one-way ANOVA followed by Tukey’s test **(C)**, and ^*^ *p* < 0.05 and ^**^ *p* < 0.01 by unpaired *t*-test **(E)**. In **(D, F)**, different letters indicate *p* < 0.05 by one-way ANOVA followed by Tukey’s test.

Endogenous GLP-1 and exogenous GLP-1R agonists are anorexigenic (20, 21). Therefore, we examined the anorexigenic ability of ISF in mice. Po administration of ISF at 30 ml/kg, but not 20 ml/kg, significantly decreased cumulative food intake during 1, 2, 3, and 6 h after injection in mice fasted overnight (**Figure 3A**). Subsequently, cumulative food intake at 24 and 48 h after po ISF returned to the control level (**Figure 3A**). In the periodical food intake, ISF (30 ml/kg, po) reduced food intake during 0–1 h after ISF injection, tended to reduce it during 1-2 h and 2-3 h, and tended to increase it during 3-6 h after ISF injection (**Figure 3B**). These results are consistent with ISF-induced gastrointestinal distension, which returned to the normal level at 6 h after injection (**Figures 1A, B** *vs.* **1D, E**). Furthermore, ISF (30 ml/kg, po), as well as control solution, did not influence saccharine preference ratio which is calculated by (consumption of saccharine solution)/(consumption of saccharine solution and water), unlike lithium chloride causing taste aversion (**Figure 3C**), suggesting that po ISF administration did not induce taste aversion.

### Anorexigenic Effect of ISF Is Partially Blocked by GLP-1R Antagonist and Systemic Capsaicin Treatment

Secretion of intestinal GLP-1 and activation of vagal afferents *via* GLP-1R signaling reportedly suppress food intake (21). Hence, we examined whether ISF decreases food intake *via* GLP-1R signaling and sensory nerves system including vagal afferents. To evaluate the involvement of GLP-1R signaling in ISF-induced anorexigenic effect, we used a GLP-1R antagonist, exendin(9-39) amide [Ex(9-39)]. Early phase of ISF-induced suppression of feeding at 1 to 2 h after injection was not significantly altered by pretreated with Ex(9-39) at 600 nmol/kg with a single shot (**Figure 3D**). However, its later phase at 3 to 6 h after injection was partially and significantly attenuated by Ex(9-39) (**Figure 3D**). We previously identified rare sugar D-allulose as a novel GLP-1 releaser and demonstrated that D-allulose suppresses food intake at 1 to 6 h after injection (21). These effects were completely inhibited both by denervation of vagal afferents and by genetic inactivation of GLP-1 receptor signaling in whole body or selectively in vagal afferents (21). In contrast, pharmacological GLP-1 receptor blockade using Ex(9-39) at 600 nmol/kg significantly attenuated D-allulose-induced anorexigenic effect in the later phase (3–6 h after injection) but not in the early phase (1–2 h after injection) (21). Therefore, it is speculated that ip administration of Ex(9-39) might not result in its substantial rise in the microcirculation in the intestine and portal where GLP-1 is sensed by vagal afferents.

Then, we used CAP-treated mice to examine the involvement of the sensory nerve system including vagal afferents in ISF-induced suppression of feeding. ISF (30 ml/kg, po) significantly decreased cumulative food intake at 1 to 3 h, however failed to suppress food intake at 6 h after injection (**Figure 3E**). In **Figure 3F**, the potencies of anorexigenic effect of ISF in intact mice (**Figure 3A**) *vs*. CAP-treated mice (**Figure 3E**) were compared. The potency of ISF at 1 h after injection was attenuated to half by CAP treatment (% of food intake; 11.0 ± 6.54% in intact mice *vs*. 52.8 ± 11.2% in CAP-treated mice, **Figure 3F**). Moreover, suppression of food intake at 6 h after po ISF injection was almost completely abolished in CAP-treated mice (**Figure 3F**). These results indicated that the early phase of ISF-induced anorexigenic effect partly depends on sensory nerves including vagal afferents, which are the necessary system to sense endogenous intestinal GLP-1 (20, 21), while the later phase of the ISF-induced suppression of feeding may be mediated largely by capsaicin-sensitive sensory nerves including vagal afferents and partly by GLP-1R signaling.

### ISF Improves Glucose Tolerance *via* Enhancing Insulin Sensitivity Mediated by GLP-1R Signaling and Capsaicin-Sensitive Sensory Nerves

**Figure 4:**
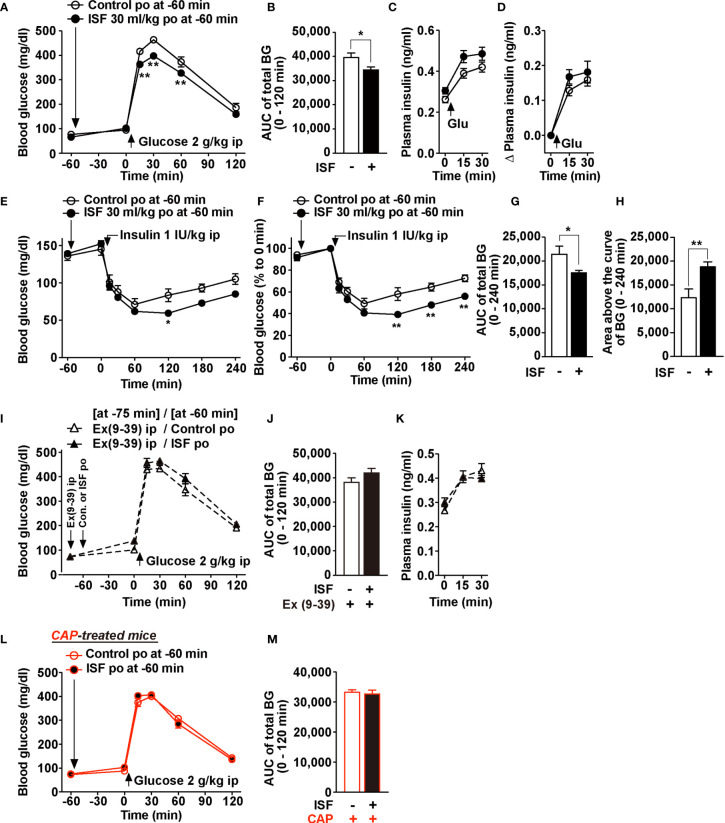
ISF improves glucose tolerance *via* GLP-1R signaling and capsaicin-sensitive sensory nerves. **(A–M)** 30 ml/kg ISF was po administered at 60 min prior to ipGTT (2 g/kg, ip) or ITT (1 IU/kg, ip). **(A–D)** Blood glucose **(A)** and plasma insulin levels **(C)** before and after administration of glucose in mice fasted overnight (16 h). Area under the curve (AUC) of total blood glucose during 0–120 min in ipGTT **(B)**. Change (Δ) in plasma insulin levels after ipGTT plotted from **(C, D)**. *n* = 11. **(E–H)** Absolute value **(E)** and relative value **(F)** of blood glucose during 0–240 min in ITT in mice fasted for 4 h. AUC of total blood glucose in E **(G)** and area above the curve of blood glucose in E **(H)**. *n* = 5–6. **(I–K)** Exendin(9-39) amide [Ex(9-39)] at 600 nmol/kg was ip injected at 75 min prior to ipGTT. Ex(9-39) pretreatment blunted the action of ISF to attenuate rises of blood glucose **(I)** and its AUC **(J)**, and also did not affect plasma insulin levels **(K)** in ipGTT. *n* = 6. **(L, M)** Po ISF failed to improve glucose tolerance in CAP-treated mice fasted overnight (16 h). *n* = 7. ^**^ *p* < 0.01 by two-way ANOVA followed by Bonferroni’s test *vs.* control **(A, E, F)**, and ^*^ *p* < 0.05 by unpaired *t*-test **(B, G, H)**.

To evaluate the effect of ISF on glucose metabolism, we performed an intraperitoneal glucose tolerance test (ipGTT) in mice fasted overnight. At 60 min before ipGTT, administration of ISF (30 ml/kg, po) that increased plasma GLP-1 concentration (**Figure 2A**) did not influence basal blood glucose levels at 0 min (**Figure 4A**). Subsequently, rises in blood glucose after ip injection of glucose (2 g/kg) were markedly suppressed at 15 to 60 min by pretreatment with ISF (**Figure 4A**), and the area under the curve (AUC) of blood glucose levels during 0 to 120 min was significantly decreased by ISF (**Figure 4B**). In contrast, plasma insulin levels before and after glucose injection were not significantly different between ISF and control solution groups (**Figures 4C, D** **)**. These results suggested that po administration of ISF improves glucose tolerance through enhancing insulin action. Therefore, to examine the effect of ISF on insulin action, insulin tolerance test was performed. Pretreatment with ISF enhanced the blood glucose lowering effect of insulin (1 IU/kg, ip) at 120 min and later (**Figures 4E, F** **)**. The AUC of blood glucose levels was significantly reduced, and the area above the curve of blood glucose was markedly increased during 0–240 min in the presence of ISF (**Figures 4G, H** **)**. These results indicate that oral administration of ISF improves glucose tolerance *via* enhancing insulin action.

Ex(9-39) (600 nmol/kg, ip) was administered with a single shot at 75 min prior to ipGTT to assess the involvement of GLP-1R signaling, followed by administration of ISF or control solution at 60 min before ipGTT (**Figure 4I**). In the presence of Ex(9-39), the effects of ISF to lower the blood glucose level and its AUC in ipGTT were completely blunted (**Figures 4I, J** **)**. Plasma insulin levels were not altered (**Figure 4K**). Furthermore, in CAP-treated mice, ISF (30 ml/kg) failed to improve glucose tolerance in ipGTT (**Figures 4L, M** **)**. These data indicate that ISF improves glucose tolerance *via* GLP-1R signaling and capsaicin-sensitive sensory nerves.

### ISF Promotes GLP-1 Secretion and Improves Glucose Tolerance in HFD-Fed Obese Mice Exhibiting Hyperglycemia

**Figure 5:**
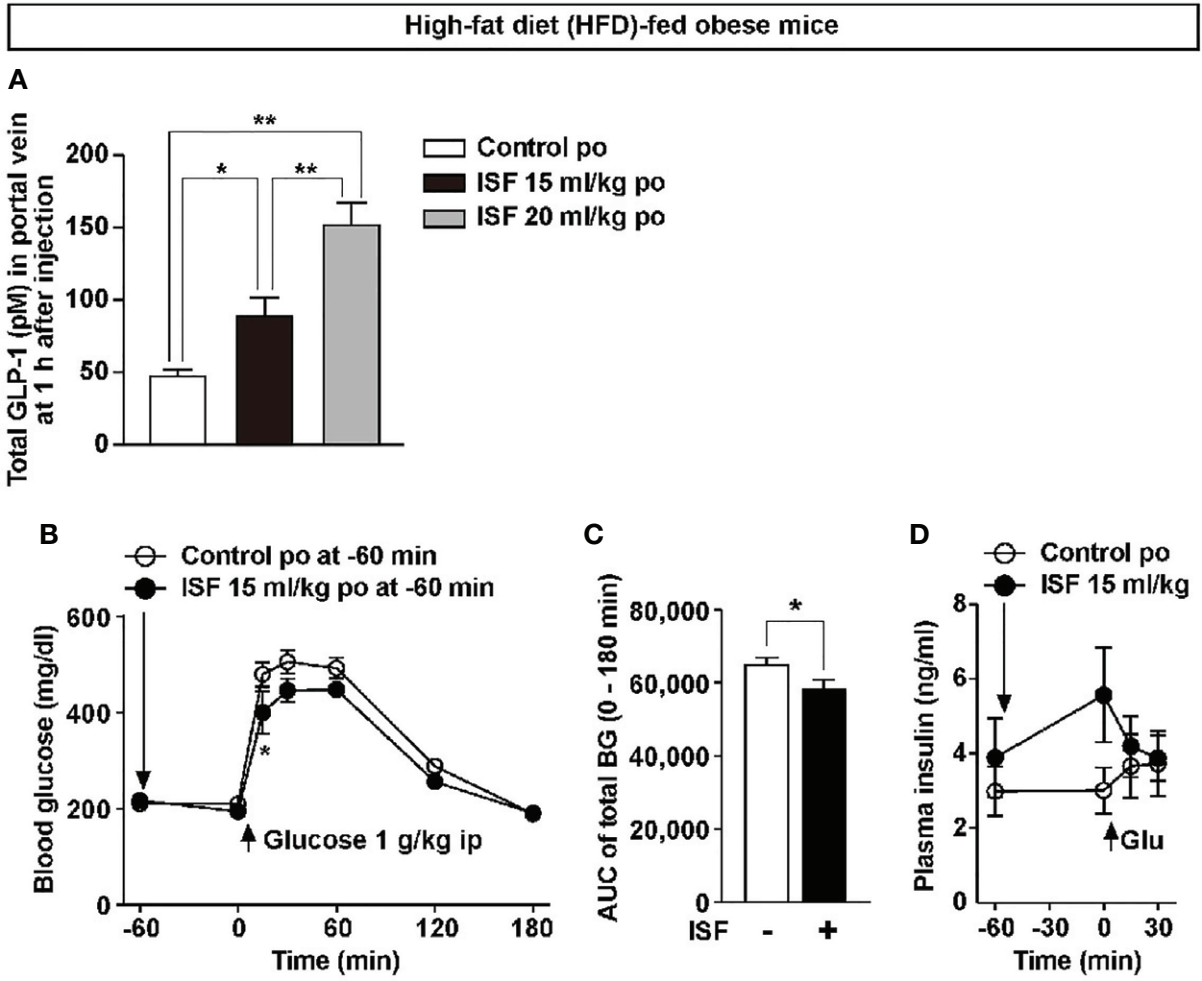
ISF promotes GLP-1 secretion in a dose-dependent manner and improves glucose tolerance in high-fat diet (HFD)-fed obese mice. **(A)** Total GLP-1 concentrations in portal vein plasma at 1 h after po ISF (15 or 20 ml/kg) in HFD-fed obese mice fasted overnight **(A)**, which exhibited obesity. *n* = 6. ^**^ *p* < 0.01, ^*^ *p* < 0.05 by one-way ANOVA followed by Tukey’s test **(A)**. **(B–D)** ISF (15 ml/kg) or control solution was po administered at 60 min prior to ipGTT (1 g/kg) in HFD-fed obese mice fasted for 4 h. Blood glucose **(B)**, its AUC **(C)** and plasma insulin **(D)**. *n* = 5–6. ^*^ *p* < 0.05 by two-way ANOVA followed by Bonferroni’s test *vs*. control **(B)**, and ^*^ *p* < 0.05 by unpaired *t*-test **(C)**.

We examined the effects of ISF on GLP-1 secretion and glucose tolerance in high-fat diet (HFD)-fed obese and diabetic mice. A previous report has indicated that the weight of the stomach in the mice aged 12 weeks and older remains constant (38). In this study, obese mice aged 20 weeks and fed HFD for 80 to 100 days, compared with the same aged lean mice fed a standard chow, showed overweight (44.5 ± 1.7 g *vs*. 29.5 ± 0.9 g, n = 6–9), but the stomach weight was slightly lower than that in lean mice (129.0 ± 2.6 g *vs*. 160.5 ± 3.3 g, n = 6–9). Therefore, we investigated whether ISF with lower dose enhances GLP-1 secretion in DIO mice. In DIO mice fed a HFD for 80 days and weighting 35.9 ± 0.7 g, po administration of ISF at 15 ml/kg (600 µl) and 20 ml/kg (800 µl) significantly increased total GLP-1 concentration in the portal vein plasma at 1 h after injection in a dose-dependent manner (**Figure 5A**). Portal plasma GLP-1 concentration at 1 h after ISF (15 ml/kg, approximately 600 µl, po) injection in DIO mice was 89.7 ± 12.0 pM, which was higher than that in lean mice administered ISF at 30 ml/kg (68.8 ± 5.9 pM, **Figure 2A** *vs*. **Figure 5A**). Furthermore, po ISF (15 ml/kg), administered 60 min before ip glucose (1 g/kg) injection suppressed the rise in blood glucose level and its AUC for 0–180 min period (**Figures 5B, C** **)**. Plasma insulin levels were not significantly different between ISF and control solution groups (**Figure 5D**).

### Subchronic Po Administration of ISF at Light Period Onset Ameliorates Hyperphagia and Visceral Obesity

**Figure 6:**
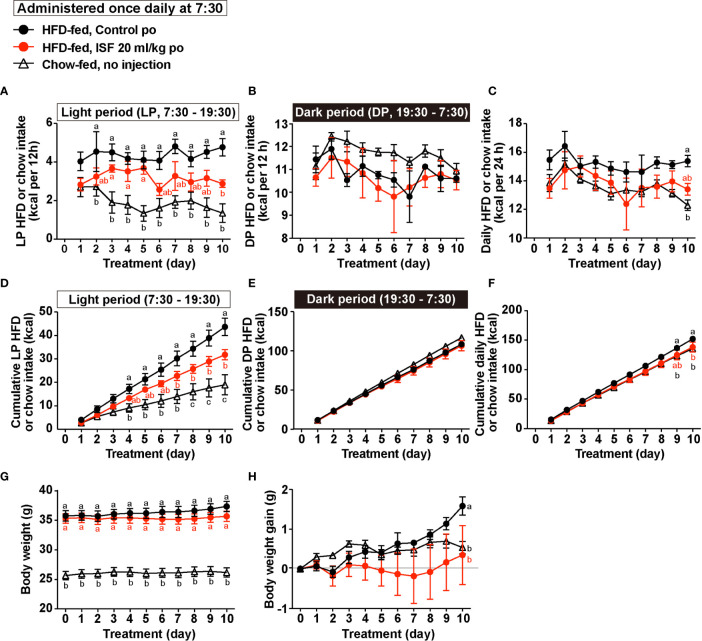
Subchronic administration of ISF (20 ml/kg/day) on hyperphagic obesity in HFD-fed mice. **(A–H)** HFD-fed mice (HFD-fed), compared to lean mice fed a standard chow (Chow-fed), exhibited light period (LP)-selective hyperphagia accompanied by daily hyperphagia (A–F). Subchronic administration once daily at LP onset (7:30) of ISF (20 ml/kg/day), compared to control solution, for 10 days in HFD-fed obese mice significantly suppressed LP HFD intake **(A, D)** and daily HFD intake **(C, F)** but not dark period (DP) HFD intake **(B, E)**, and lowed body weight gain **(H)**. *n* = 5. Different letters indicate *p* < 0.05 by two-way ANOVA followed by Tukey’s test **(A–H)**.

HFD-fed obese mice show the impairment of diurnal feeding rhythm with the light period (LP) hyperphagia (39), and correction of the LP hyperphagia decreases body weight and ameliorates visceral obesity and diabetes (21). In the present study, cumulative food intake in LP (7:30–19:30) in obese mice fed a HFD for 8 weeks was approximately doubled compared with lean mice fed a standard chow, whereas there was no difference in food intake during dark period (DP, 19:30–7:30, **Figures 6A, B, D, E** **)** between obese and lean mice. Thus, DIO mice displayed LP-specific hyperphagia accompanied by daily hyperphagia (**Figures 6C, F** **)**. Hence, we examined whether subchronic administration of ISF daily at LP onset for 10 days ameliorates LP hyperphagia and obesity in HFD-fed obese mice.

Subchronic po administration of ISF (20 ml/kg) daily at the LP onset (7:30) partially suppressed LP hyperphagia and significantly decreased cumulative LP food intake for 10 days (**Figures 6A, D** **)**. On the other hand, the ISF did not alter DP food intake per day and for 10 day (**Figures 6B, E** **)**. As a result, the daily food intake and cumulative food intake for 10 days in DIO mice administered ISF were reduced to the levels close to those of lean mice (**Figures 6C, F** **)**, and the body weight gain was inhibited by subchronic ISF administration (**Figures 6G, H** **)**.

**Figure 7:**
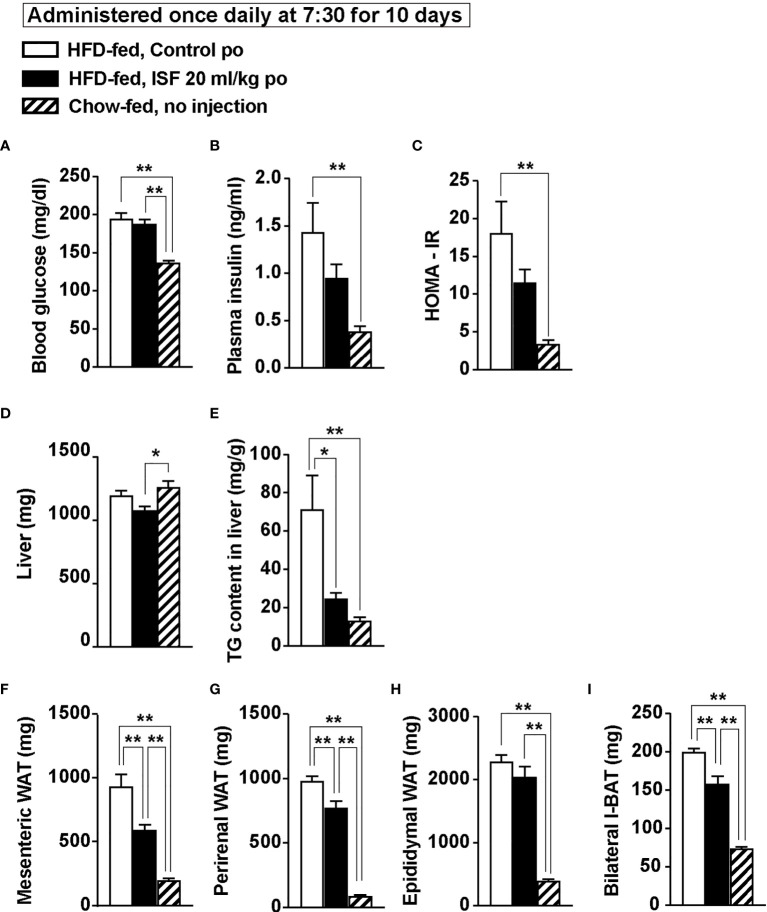
Subchronic po administration of 20 ml/kg ISF at LP onset reduced hepatic triacylglycerol (TG) content and ameliorates visceral obesity on Day 11 in HFD-fed obese mice. **(A–C)** Subchronic administration of ISF (20 ml/kg/day) did not change fasting glucose **(A)**, but tended to attenuate hyperinsulinemia **(B)** and insulin resistance **(C)** in DIO mice on Day 11. **(D–I)** Subchronic ISF treatment did not affect liver weight **(D)**, but markedly reduced hepatic TG content **(E)** and weight of visceral white adipose tissues (WAT) including mesenteric, perirenal and epididymal WAT **(F–H)** on Day 11. ISF also reduced bilateral interscapular brown adipose tissues (I-BAT, **I**). *n* = 5. ^*^ *p* < 0.05 and ^**^ *p* < 0.01 by one-way ANOVA followed by Tukey’s test **(A–I)**.

At Day 11 after treatment with ISF for 10 days, blood component and weight of organs were measured. DIO mice showed hyperglycemia and hyperinsulinemia in comparison with lean mice (**Figures 7A, B** **)**. ISF treatment did not markedly affect hyperglycemia, but partially improved hyperinsulinemia and insulin resistance expressed by HOMA-IR (**Figures 7A–C**). Furthermore, subchronic ISF significantly decreased liver triacylglycerol (TG) content without altering weight of liver (**Figures 7D, E** **)** and significantly reduced the weight of mesenteric and perirenal white adipose tissues (WAT, **Figures 7F, G** **)** and interscapular brown adipose tissues (I-BAT, **Figure 7I**) but not the weight of epididymal WAT (**Figure 7H**). Additionally, ISF increased expressions of lipid metabolism-related genes in epididymal WAT: peroxisome proliferator-activated receptor *γ* (Ppar*γ*), adipose triglyceride lipase (Atgl), and hormone-sensitive lipase (Hsl) (**Supplementary Figures 2A–C**), while protein expression level of uncoupling protein-1 (UCP-1) in I-BAT was unaltered (**Supplementary Figures 2D, E** **)**. These results indicate that subchronic ISF administration at LP onset corrects diurnal feeding rhythm and hyperphagia, which might additionally contribute to improve insulin resistance and fat accumulation.

## Discussion

In the present study, we showed that temporary gastrointestinal distension by po administration of ISF stimulated intestinal GLP-1 secretion and activated “vagal afferents–brain” axis, thereby suppressing food intake and promoting glucose tolerance. ISF improved glucose tolerance primarily *via* promoting insulin action rather than insulin secretion in lean and DIO-obese mice. Pretreatment with GLP-1R antagonist and denervation of capsaicin-sensitive sensory nerves substantially counteracted the actions of ISF to inhibit food intake and to improve glucose tolerance. In HFD-fed obese mice showing arrhythmic feeding, subchronic po administration of ISF once a daily at LP onset (7:30) for 10 days restored diurnal feeding rhythm by suppressing LP hyperphagia and suppressed fat accumulation in visceral adipose tissue and liver. These results indicate that subchronic ISF, possibly *via* periodical gastrointestinal distension, ameliorates arrhythmic overeating and obesity *via* GLP-1 release and activation of capsaicin-sensitive sensory nerves including vagal afferents.

Gastric distension is thought to be an important signal that promotes meal termination (22). In contrast, previous reports showed that distension of the stomach using a balloon or bolus saline is not sufficient to induce satiation by itself (29, 40). In addition, slowing of gastric emptying and hence increased gastric distension with the use of GLP-1 receptor agonist (lixisenatide) are shown to be unrelated to energy intake in humans (41). In the present study, we have demonstrated that distension of gastrointestinal tract by ISF suppressed food intake without aversive behavior. The stomach is extensively innervated by numerous visceral sensory nerves including mechanosensitive vagal afferents (12, 42). Gastric distension using a balloon activates vagal afferents innervating the stomach (27) and evokes anorectic effect producing aversion (28). A recent study showed that intestinal distension suppressed food intake *via* activation of vagal afferents innervating the intestine and inhibition of orexigenic AgRP neurons in the arcuate nucleus of the hypothalamus (43). However, the mechanisms of how the gastric and/or intestinal expansion activates vagal afferent neurons remain unclear. The present study has shown that peroral administration of ISF expanded the upper gastrointestinal tract including not only the stomach but also the duodenum and ileum, thereby inducing GLP-1 release and activating vagal afferents. We previously reported that oral administration of D-allulose, a rare sugar, stimulates GLP-1 release from the intestine, which in turn directly activates vagal afferent neurons that express GLP-1R, thereby decreasing food intake (21). Taken together, the gastrointestinal distension by ISF, especially distension of intestine, might suppress food intake through GLP-1 release and consequent activation of vagal afferent activation. However, the mechanism underlying the release of GLP-1 by gastrointestinal distension remains to be elucidated, and hence further study is required. Furthermore, in addition to GLP-1, the involvement of gastrointestinal hormones CCK and PYY_3–36_, which suppress food intake *via* vagal afferents (15), is required to be examined in future.

The early phase of ISF-induced anorexigenic effect (1 to 2 h after injection) was not attenuated by GLP-1R antagonist and denervation of capsaicin-sensitive sensory nerves. There are at least two possible pathways for this early phase of the inhibition feeding. First, capsaicin-insensitive myelinated A-fiber, which senses mechanical stretch and tension, might be involved in the anorexigenic effect by ISF. However, in this study, pERK1/2 expression, a neural activation marker, was not detected in NGs and NTS in CAP-treated mice. Phosphorylation of ERK 1/2 is transient, while the time course of activation of sensory neurons depends on the type of neurons and stimulus including mechanical *vs*. chemical stimuli (44, 45). The expression of pERK1/2 was induced at 2 min after electrical stimulation in dorsal root ganglion neurons (44) and induced at 15 to 30 min after administration of gastrointestinal/pancreatic hormones or its secretagogues in NGs and NTS (21, 36). In this study, we examined the expression of pERK1/2 in NG and NTS at 30 min after administration of ISF. Therefore, further study is needed to examine pERK1/2 expression at the optimal timing after mechanical stimulation of vagal afferents. Vagal afferents partly project to AP (11, 46), and ISF activates AP in both intact and CAP-treated mice in this study (**Supplementary Figure 1**). Hence, the gastrointestinal distension by ISF might activate AP *via* vagal afferent CAP-insensitive mechanosensitive A-fiber. As the second possibility, gastrointestinal distension by ISF might activate AP *via* gut-derived humoral factors. AP is anatomically located outside the blood–brain barrier (47) and could sense peripheral signals. Serotonin, which abounds in enterochromaffin cells, is released in response to a wide variety of stimuli including mechanical distension of the intestine (48, 49). Peripheral administration of serotonin reportedly decreases food intake *via* direct action on AP but not vagal afferents (50–52). Thus, ISF-induced gastrointestinal distension might activate AP *via* humoral factors such as serotonin, thereby acutely suppressing feeding.

Eating vegetables, the food with low energy-density and high-volume, before meal reduces postprandial glucose and insulin (25, 53). Intake of salad and/or dietary fiber reportedly induces gastric distension and delays gastric emptying and digestion/absorption (53, 54). Furthermore, previous dietary interventions indicate that GLP-1 release slows gastric emptying and attenuates elevation of blood glucose and insulin at postprandial phase (55, 56). The present study suggests that gastrointestinal distension by ISF enhances insulin sensitivity *via* GLP-1 secretion and activation of sensory nerves in lean and diabetic DIO mice. We previously demonstrated that intestinal GLP-1 release by the rare sugar D-allulose enhances insulin action *via* GLP-1R signaling in both lean and DIO mice (21). Taken together, the present study suggests that gastrointestinal distension before meal might suppress postprandial rises in blood glucose and insulin through releasing GLP-1 and activating vagal afferents. In this study, however, we did not measure plasma level of glucose-dependent insulinotropic polypeptide (GIP), a gut hormone that regulates glucose tolerance through insulin secretion (57). Therefore, other gastrointestinal hormones including GIP could be involved in the regulation of glucose metabolism by ISF.

Obese model mice (HFD-fed DIO mice, histamine H_1_ receptor knockout mice) and rats (Zucker obese rat with a leptin receptor mutation) exhibit LP-specific hyperphagia and disruption of diurnal feeding rhythm (58–60), and time-restricted feeding schedule to avoid feeding at light phase without reducing daily caloric intake prevented obesity (39, 59). Previous reports showed that intracerebroventricular administration of oxytocin or oral administration of rare sugar D-allulose (GLP-1 releaser) at LP onset, but not DP onset, ameliorated arrhythmic overeating, obesity, and diabetes in DIO mice or rats (21, 61). There results indicate that not only hyperphagia but also arrhythmic feeding cause obesity, and administration of anorexigenic factors at LP onset is more effective to ameliorate arrhythmic feeding and obesity. In this study, we demonstrated that GLP-1 release by ISF-induced gastrointestinal distension at LP onset also corrects diurnal feeding rhythm and obesity, indicative of the importance of GLP-1 release at LP onset for the treatment of arrhythmic overeating and obesity.

In the future, the studies focusing on the central nerve system underlying the regulation of feeding and glucose metabolism by ISF-induced gastrointestinal distension and GLP-1 release will be important. In a recent study using DREADD system, activation of the vagal afferent nerves that express GLP-1 receptors suppresses food intake and the neural activity of NPY/AgRP neurons in the arcuate nucleus of hypothalamus (43). Therefore, NPY/AgRP neurons in the hypothalamus could be involved in the ISF-induced anorexigenic effect.

Bariatric surgery such as Roux-en-Y gastric bypass (RYGB) and sleeve gastrectomy provides effective therapy for hyperphagia, obesity, and type 2 diabetes. These therapeutic effects are associated with the excessive gastrointestinal distension due to reduced stomach capacity and with changes in gastrointestinal hormones in circulation including GLP-1 and PYY (62–64). Here, we demonstrate that gastrointestinal distension after ISF intake ameliorates hyperphagia and obesity *via* the mechanism similar to that of bariatric surgery. A bulky diet rich in vegetables is used as an effective diet therapy in diabetic and obese subjects. Since bulky diet is likely to induce gastrointestinal distension, it might involve GLP-1 release and activation of vagal afferents. GLP-1R agonists are used worldwide as a major medicine to treat type 2 diabetes and obesity. Therefore, the diet and formulation that distend gut and release GLP-1 may provide a new evidence-based diet therapy to treat arrhythmic overeating, obesity, and diabetes.

## Data Availability Statement

The original contributions presented in the study are included in the article/**Supplementary Material**. Further inquiries can be directed to the corresponding author.

## Ethics Statement

The animal study was reviewed and approved by the Committee for Animal Research of the Kyoto Prefectural University (permission no. KPU020209, KPU020210, and KPU020213).

## Author Contributions

KO, YO, TA, TY, and YI designed and conceived the experiments. KO, YO, CY, and YI performed the experiments. KO, TY, and YI wrote the manuscript. All authors contributed to data interpretation and the drafting of the manuscript. All authors contributed to the article and approved the submitted version.

## Funding

This study was supported by the grant for Taisho Pharmaceutical Co., LTD.

## Conflict of Interest

YO, CY, and TA were employed by Taisho Pharmaceutical Co., LTD. YI and TY declare that this study received funding from Taisho Pharmaceutical Co., LTD. The funder provided ISF test solution and was partly involved in designing and performing the experiments, but was not involved in data collection, making figures, statistical analysis, and manuscript preparation.

The remaining author declares that the research was conducted in the absence of any commercial or financial relationships that could be construed as a potential conflict of interest.

## Supplementary Material

Supplementary Figure 1 Po administration of ISF activates area postrema (AP) in intact mice and capsaicin-treated mice. **(A–E)** Immunostaining of pERK1/2-immunoreactive (IR) in the AP was performed at 30 min after po administration of ISF (30 ml/kg) or control solution (30 ml/kg) in intact and capsaicin (CAP)-treated mice. We used an imaging analysis system (NIH Image/ImageJ 1.50a) to average the pERK1/2-IR fluorescence intensity per unit area in the AP. Po administration of ISF (30 ml/kg) markedly increased pERK1/2-IR fluorescence intensity in AP in both intact **(A, B, E)** and CAP-treated mice **(C–E)**. **p* < 0.05 and ***p* < 0.01 by one-way ANOVA followed by Tukey’s test. Scale bar indicates 100 µm.

Supplementary Figure 2 Expression of lipid metabolism-related genes in white adipose tissue (WAT) and uncoupling protein-1 in brown adipose tissue (BAT) in DIO mice subchronically treated with ISF. **(A–C)** Messenger RNA expression levels of the genes involved in lipid metabolism (**A**; Ppar*γ*, **B**; Atgl, **C**; Hsl) in epididymal WAT derived from DIO mice treated with ISF at LP onset for 10 days. **(D, E)** Uncoupling protein-1 (UCP-1) protein expression in interscapular BAT. *n* = 5. **p* < 0.05 and ***p* < 0.01 by unpaired *t*-test.
