# Supplementary material for: Gastrointestinal Distension by Pectin-Containing Carbonated Solution Suppresses Food Intake and Enhances Glucose Tolerance via GLP-1 Secretion and Vagal Afferent Activation
Source: Front Endocrinol (Lausanne). 2021 Jun 8;12:676869. doi: 10.3389/fendo.2021.676869 (PMC8217665; doi:10.3389/fendo.2021.676869)
Supplement: Supplementary Figure 1 — Po administration of ISF activates area postrema (AP) in intact mice and capsaicin-treated mice. (A–E) Immunostaining of pERK1/2-immunoreactive (IR) in the AP was performed at 30 min after po administration of ISF (30 ml/kg) or control solution (30 ml/kg) in intact and capsaicin (CAP)-treated mice. We used an imaging analysis system (NIH Image/ImageJ 1.50a) to average the pERK1/2-IR fluorescence intensity per unit area in the AP. Po administration of ISF (30 ml/kg) markedly increased pERK1/2-IR fluorescence intensity in AP in both intact (A, B, E) and CAP-treated mice (C–E). *p < 0.05 and **p < 0.01 by one-way ANOVA followed by Tukey’s test. Scale bar indicates 100 µm. [file DataSheet_1.docx]

**Supplementary Methods**

**Quantitative PCR analysis of the genes related to lipid metabolism**

The part of epididymal white adipose tissue removed from obese mice was minced and immediately soaked in RNAlater (Thermo Fisher Scientific). Total RNA was extracted with RNeasy Plus Universal Mini Kit (QIAGEN) according to the manufacturer's protocol. For reverse transcription, 100 ng of total RNA was transferred to the reaction with ReverTra Ace qPCR RT Master Mix (TOYOBO, Osaka, Japan). Quantitative real-time PCR was performed using THUNDERBIRD SYBR qPCR MIX (TOYOBO) on a Step One Plus Real Time PCR System (Thermo Fisher Scientific). Relative amounts of mRNA were calculated by the standard curve methods, and the expression of targeted mRNAs (Ppar*γ*, *Atgl* and Hsl) were normalized to *β-actin* mRNA levels. Primer sequences were as follows: 5′-GGAGCCTAAGTTTGAGTTTGCTGTG-3′ and 5′-TGCAGCAGGTTGTCTTGGATG-3′ (Ppar*γ*); 5′-GTGAAGCAGGTGCCAACATTATTG-3′ and 5′-AAACACGAGTCAGGGAGATGCC-3′ (*Atgl*); 5′-TCCTGGAACTAAGTGGACGCAAG-3′ and 5′-CAGACACACTCCTGCGCATAGAC-3′ (Hsl); 5′-CATCCGTAAAGACCTCTATGCCAAC-3′ and 5′-ATGGAGCCACCGATCCACA-3′ (*β-actin*)

**Western blot analysis of UCP1 in brown adipose tissue**

Interscapular brown adipose tissue was homogenized in RIPA buffer (Nacalai Tesque, Kyoto, Japan) with EDTA and Halt™ Protease and Phosphatase Inhibitor Cocktail (Thermo Fisher Scientific) and left to stand for 1 hour on ice. After centrifugation at 10,000 × g for 10 min at 4 °C, the supernatant was collected. Protein concentrations were determined by BCA Protein Assay (Takara Bio, Shiga, Japan). Protein lysates were diluted with Sample Buffer Solution with Reducing Reagent (6x) for SDS-PAGE (Nacalai Tesque) and incubated at 95 °C for 5 min. Protein (5 µg) was separated using 10% SDS-PAGE and transferred to polyvinylidene fluoride membranes.

Membranes were blocked in Blocking One (Nacalai Tesque), then incubated overnight at 4°C with primary antibodies: anti-UCP1 (Merck, Cat. 662045, 1:1000); anti-β-Tubulin (Cell Signaling Technology, Cat. 2146, 1:1000). After wash, membranes were incubated with horseradish peroxidase (HRP)-conjugated IgG secondary antibody (Cell Signaling Technology, Cat. 7074, 1:4000 for UCP1, 1:2000 for β-Tubulin) for 1 hour at room temperature. Bands were detected with Pierce™ ECL Western Blotting Substrate (Thermo Fisher Scientific) using ChemiDoc XRS (Bio-Rad).

The intensity of bands was analyzed using Image Lab Software (Bio-Rad). UCP1 protein expression were normalized to β-Tubulin protein levels.

**Supplementary Figure 1.** Po administration of ISF activates area postrema (AP) in intact mice and capsaicin-treated mice. **(A-E)** Immunostaining of pERK1/2-immunoreactive (IR) in the AP was performed at 30 min after po administration of ISF (30 ml/kg) or control solution (30 ml/kg) in intact and capsaicin (CAP)-treated mice. We used an imaging analysis system (NIH Image/ImageJ 1.50a) to average the pERK1/2-IR fluorescence intensity per unit area in the AP. Po administration of ISF (30 ml/kg) markedly increased pERK1/2-IR fluorescence intensity in AP in both intact (A, B, E) and CAP-treated mice (C, D, E). ^*^*p* < 0.05 and ^**^*p* < 0.01 by one-way ANOVA followed by Tukey’s test. Scale bar indicates 100 µm.

**Supplementary Figure 2.** Expression of lipid metabolism-related genes in white adipose tissue (WAT) and uncoupling protein-1 in brown adipose tissue (BAT) in DIO mice subchronically treated with ISF. **(A-C)** Messenger RNA expression levels of the genes involved in lipid metabolism (A; Ppar*γ*, B; Atgl, C; Hsl) in epididymal WAT derived from DIO mice treated with ISF at LP onset for 10 days. **(D, E)** Uncoupling protein-1 (UCP-1) protein expression in interscapular BAT. *n* = 5. ^*^*p* < 0.05 and ^**^*p* < 0.01 by unpaired *t*-test.
